# The Investigation of the Effect of Filler Sizes in 3D-BN Skeletons on Thermal Conductivity of Epoxy-Based Composites

**DOI:** 10.3390/nano12030446

**Published:** 2022-01-28

**Authors:** Zhengdong Wang, Tong Zhang, Jinkai Wang, Ganqiu Yang, Mengli Li, Guanglei Wu

**Affiliations:** 1 School of Mechanical and Electrical Engineering, Xi’an University of Architecture and Technology, Xi’an 710055, China; zt@xauat.edu.cn (T.Z.); jkwang@xauat.edu.cn (J.W.); yangganqiu@xauat.edu.cn (G.Y.); limengli@xauat.edu.cn (M.L.); 2 Shaanxi Key Laboratory of Nano Materials and Technology, Xi’an University of Architecture and Technology, Xi’an 710055, China; 3 State Key Laboratory of Bio-Fibers and Eco-Textiles, Institute of Materials for Energy and Environment, College of Materials Science and Engineering, Qingdao University, Qingdao 266071, China

**Keywords:** 3D boron nitride skeleton, size effect, epoxy composites, through-plane thermal conductivity

## Abstract

Thermally conductive and electrically insulating materials have attracted much attention due to their applications in the field of microelectronics, but through-plane thermal conductivity of materials is still low at present. In this paper, a simple and environmentally friendly strategy is proposed to improve the through-plane thermal conductivity of epoxy composites using a 3D boron nitride (3D-BN) framework. In addition, the effect of filler sizes in 3D-BN skeletons on thermal conductivity was investigated. The epoxy composite with larger BN in lateral size showed a higher through-plane thermal conductivity of 2.01 W/m·K and maintained a low dielectric constant of 3.7 and a dielectric loss of 0.006 at 50 Hz, making it desirable for the application in microelectronic devices.

## 1. Introduction

With the rapid development of 5G, artificial intelligence and the Internet of Things, there is a significant demand for thermally conductive and electrically insulating materials for microelectronic devices, which are suffering from elevated operation temperature [1,2,3,4]. This is mainly attributed to the fact that the heat generated from the chip during the operation of microelectronic devices cannot be quickly transferred to the cooling equipment due to a layer of thermal interface materials (TIMs). The main function of TIMs is to fill the gap between the microelectronic device and the radiator fin so that the interface thermal resistance will be reduced [5]. Polymers, such as epoxy resin or silicone rubber, are commonly used as TIMs due to their superior adhesiveness, thermal stability and electrical insulation [6,7]. However, their low TC values (below 0.3 W/m·K) cannot satisfy the needs of microelectronic devices. Therefore, TIMs with excellent through-plane thermal conductivity are urgently needed, which can transfer heat timely to the radiator fin and subsequently transmit heat outside of the device.

The composite strategy has been deemed as the most effective method to enhance thermal conductivity by incorporating ceramic fillers, such as AlN [8,9,10], Al_2_O_3_ [11,12,13], Si_3_N_4_ [14] and BN [15,16]. Especially for BN with a similar layered structure of graphite, it has attracted great interest due to its superior thermal conductivity (about 600 W/m·K in the plane direction) and wide band gap [17,18,19,20]. Therefore, the incorporation of BN into polymer has placed great significance on increasing thermal conductivity. Nevertheless, the through-plane thermal conductivity of BN-based composites prepared by a traditional blending method is much inferior to that of in-plane orientation. In this regard, some strategies have been developed to enhance the through-plane thermal conductivity of polymer composite materials. One strategy is to build a three-dimensional network skeleton. In this structure, fillers can form interconnected thermal conductivity paths, giving rise to a reduction in interface thermal resistance in polymer composites [6,21,22,23]. For instance, Chen et al. [24] used nanocellulose as the connecting intermediate to construct a three-dimensional network framework with interconnected BNNS and then infiltrated the epoxy resin solution into the framework, thus obtaining a nanocomposite with a thermal conductivity of 3.13 W/m·K. However, the phonon conduction was weakened due to the random distribution of BNNS in the framework, which made it difficult to further improve the thermal conductivity. In order to address the problem, the other strategy was developed to build the oriented structure of 1D or 2D fillers, making a high thermal conductivity ordered crystal plane arrangement and promoting directional phonon conduction. As a result, the interface thermal resistance was reduced to a low filler content [25,26,27,28,29]. However, in the above-mentioned oriented structures, there is a lack of research on the effect of filler sizes on thermal conductivity. The fillers with a small lateral size will lead to more interfaces, leading to more phonon scattering. In addition, intrinsic thermal conductivity of 2D fillers will increase with the increasing lateral size on the basis of theoretical research results [30,31]. Unfortunately, the thickness of the large-size 2D filler is also greater. In addition, the thicker 2D filler will lead to a weak interface force with a polymer matrix, resulting in the deterioration of thermal transport performance. Therefore, the sizes and aspect ratios of 2D fillers are important for improving the thermal conductivity of polymer composites and practical engineering applications.

In this work, we prepared a 3D network skeleton with aligned BN in an epoxy-based composite and investigated the correlation between the size and aspect ratio of BN and thermal conductivity. Additionally, we proposed a technique for permeating the epoxy resin into the 3D-BN skeleton under vacuum conditions. This method can ensure that the 3D-BN network frame is fully filled with the epoxy solution to reduce the voids in the composites. Another important preparation process is to observe the sample along the Z-axis to obtain directional BN across the plane. The epoxy composite with a bigger BN size showed a higher through-plane thermal conductivity of 2.01 W/m·K and lower dielectric constant and dielectric loss, which is suitable for the application of microelectronic devices. Moreover, the preparation of the polymer composites with 3D-BN is simple, eco-friendly and scalable, and the TIM is promising for practical applications in microelectronic packaging in the next generation of electronic devices.

## 2. Experimental Section

### 2.1. Materials

Bisphenol A-epoxy resin (DGEBA, E-828) was purchased from HEXION. Methyl tetrahydrophthalic anhydride (MTHPA) and N,N-dimethylbenzylamine (BDMA) were supplied from Aladdin Industrial Corporation (Shanghai, China), which were employed as curing agent and accelerator, respectively. Isopropyl alcohol (99%) was purchased from Aladdin Industrial Corporation (Shanghai, China). BN platelets in various sizes were supplied by Dandong Chemical Research Institute Limited Company (Dandong, China), and the larger ones, middle ones and smaller ones were abbreviated as LBN, MBN and SBN, respectively.

### 2.2. Preparation of 3D-BN Framework and 3D-BN/Epoxy Composites

**Figure 1:**
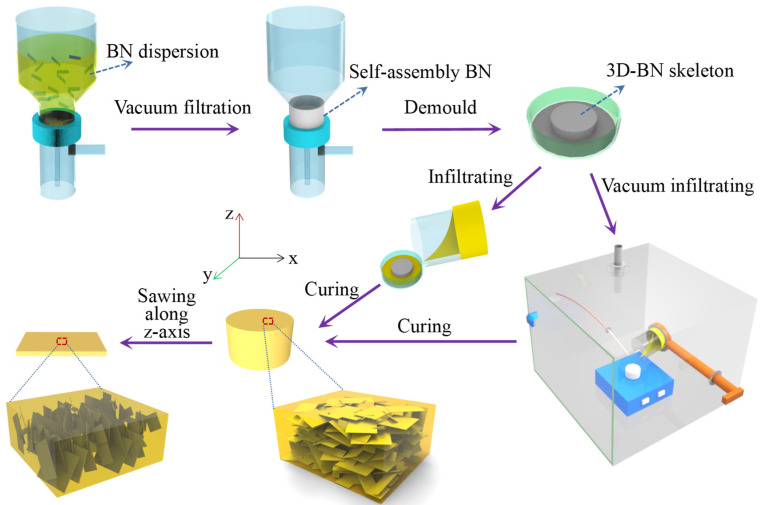
Sketch drawing on preparation process of 3D-BN/epoxy composites.

The 3D-BN framework was prepared by a typical vacuum filter self-assembly process [32]. In brief, 1 g BN platelets were dispersed in 200 mL IPA solvent by sonicating for 1 h in an ultrasonic bath to obtain a homogeneous BN/IPA suspension. The block BN cake with a height of 1 cm was obtained by BN/IPA suspension filtration. And then the filter paper below in the cake was removed after drying for overnight at 60 °C. To prepare epoxy composites, the mixture of epoxy monomers, hardeners and accelerators was dispersed by using a planetary mixer (Thinky, Waltham, Massachusetts, USA). Then, the mixed solution was immersed into the 3D-BN frame in the atmospheric and vacuum environment respectively. The air bubbles in the mixture of BN and epoxy solution were removed by vacuum system under 60 °C for 120 min. After curing processing of 120 min at 100 °C and subsequent 600 min at 150 °C, the sample was peeled from the mould. The sample was sawed along the Z-axis to obtain a slice with oriented BN in the through-plane direction. The samples obtained by incorporating 3D-LBN in the atmospheric and vacuum environment were named as A-3D-LBN/epoxy and V-3D-LBN/epoxy, respectively. For MBN and SBN, abbreviations were set in a similar way. Figure 1 elucidates the preparation process of BN/epoxy composites.

### 2.3. Characterization

Thermal diffusivity (*α*) and specific heat (*C*_p_) were tested via laser flash analyzer (LFA 467 Nanoflash, NETZSCH, Germany)). Densities (*ρ*) of specimens were estimated by measuring their volume and mass. TC values of specimens were obtained by a typical equation: *TC* = *α* × *C*_p_ × *ρ*. The specimen was sliced into a square sheet with 10 mm in side length and 1 mm in thickness. Subsequently, graphite coating was deposited onto the surface of samples. For dielectric measurements, permittivity and dielectric loss were measured via dielectric analyzer (CONCEPT80, Nov Tech Co, Montabaur, Germany)). Gold coating was deposited onto the surface of samples via an auto sputtering device. Thermal gravimetric analyzer and differential scanning calorimeter (200F3, NETZSCH, Germany)) were performed at the heat rate of 10 °C/min in air and nitrogen atmosphere, respectively. Surface morphology and microstructure of fillers and their composites were investigated by a field emission SEM (FEI QUANTA F250, Waltham, MA, USA) and X-ray diffractometer (D2 PHASER, Bruker). The surface temperature images of the samples were obtained by an infrared thermal imager (Fluke TiS 65/60, Everett, WA, USA). The dynamic mechanical analysis was performed by a dynamic mechanical analyzer (DMA Q800, TA Instruments, Shanghai, China) [33]. Specimen was sliced into the size of 40 mm × 10 mm × 1 mm, which was loaded onto a flexural mode at the heat rate of 5 °C/min at 6.28 rad/s under air atmosphere.

## 3. Results and Discussion

### 3.1. Morphology and Microstructure Analysis of BN, 3D-BN/Epoxy Composites

**Figure 2:**
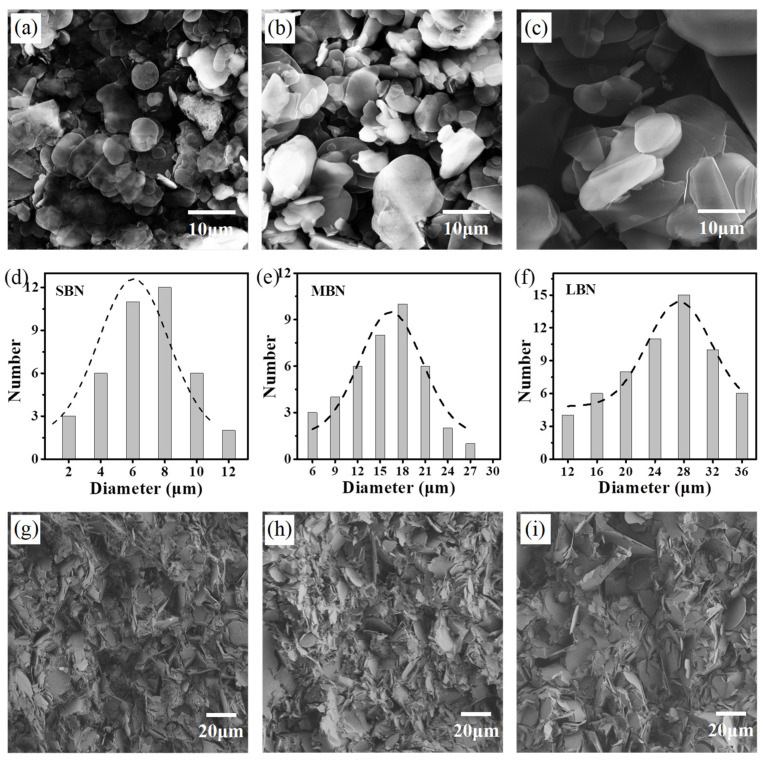
Surface morphology of BN platelets (**a**) SBN, (**b**) MBN and (**c**) LBN from SEM images. (**d**–**f**) the average lateral sizes of BN platelets via Gaussian statistics. SEM images of the epoxy composites with different filler sizes (**g**) V-3D-SBN/epoxy, (**h**) V-3D-MBN/epoxy and (**i**) V-3D-LBN/epoxy.

Figure 1 shows the process of preparing 3D-BN/epoxy composites using filter self-assembly and subsequent vacuum casting, followed by sawing along the *Z*-axis direction. Finally, the epoxy composites with vertically-oriented BN platelets (perpendicular to the planar direction of sample) were obtained. In these kinds of epoxy composites, the BN skeleton via vacuum filtration self-assembly has a three-dimensional network with interconnected structures. Three kinds of BN platelets (denoted as SBN, MBN and LBN based on the size from small to large) were used. The morphology and size of the BN platelets are exhibited in Figure 2a–c and have a similar flake-like structure and different lateral sizes, and the LBN platelets have a larger lateral size (more than 30 μm). The lateral sizes and thicknesses of different BN platelets were measured and marked by SEM and counted by Gaussian statistical distribution, as shown in Figure 2 and Figure S1 and Figure S2, respectively. The statistical sizes of SBN, MBN and LBN are compared in Figure 2d–f, and the average lateral sizes of them are 6.1 μm, 16.4 μm and 27.5 μm, respectively. However, their average thicknesses are close, and therefore LBN showed the highest ratio of lateral size and thickness compared to those of SBN and MBN. The 2D materials such as BN with a higher ratio of lateral size and thickness in the solvent will preferentially form a horizontal alignment via self-assembly under gravity [34].

The surface morphology and structure of V-3D-BN/epoxy composites prepared by various methods were compared and studied. As revealed by the SEM images in Figure 2, the fractured structure of the V-3D-BN/epoxy composites with different transverse sizes had a similar distribution of BN due to the same preparation processing by filtrating self-assembly and casting. These epoxy composites showed a distinct orientation of BN in 3D skeletons along the *Z*-axis (through-plane) direction. In addition, during the vacuum casting process, few voids were observed in these epoxy composites due to the complete removal of bubbles in the vacuum environment, as shown in Figure 2g–i. Moreover, the amount of BN in the 3D network skeletons was significantly different, and the V-3D-LBN/epoxy composite show a bigger transverse size in Figure 2i. Moreover, we found that the composite prepared by atmospheric pressure casting had more voids, and the partial 3D-BN networks were easier to destroy in the process of degassing. In order to solve this problem, the technique of vacuum casting was developed; the voids were much fewer, and the 3D network frameworks were significantly maintained. The 3D-BN networks with interconnected structures and a partially oriented distribution were beneficial for forming thermal transport paths on the oriented direction [35,36].

### 3.2. Thermal Conductivity

In general, the thermal conductivity of inorganic–organic composites is basically determined by the structure, dispersion, loading capacity, surface properties of inorganic materials and the thermal resistance between the interfacial filler and the polymer matrix [37,38,39,40,41].

**Figure 3:**
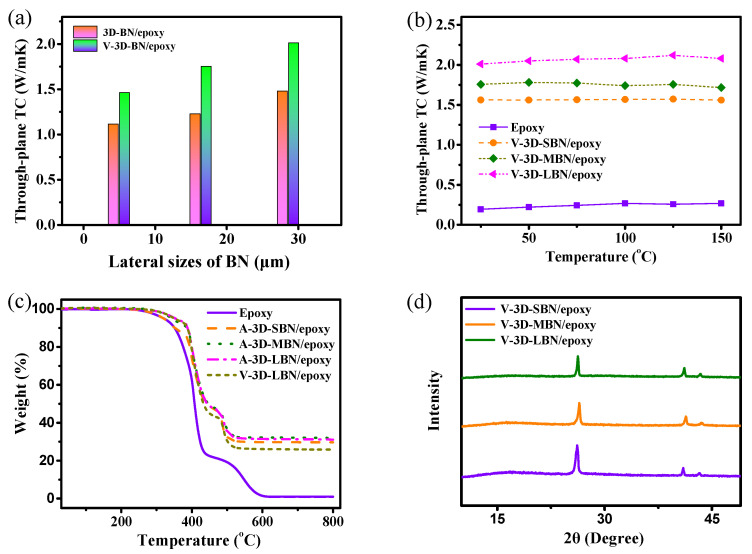
(**a**) Through-plane TC and (**b**) TGA curves of the 3D-BN/epoxy composites. (**c**) Through-plane TC at various temperature and (**d**) XRD patterns of the epoxy and V-3D-BN/epoxy composites.

Figure 3a exhibits the through-plane TC values of epoxy and its composites with diverse BN sizes, and the TC of composites prepared by filtration self-assembly and casting under an atmospheric and a vacuum environment were compared. Remarkably, the composites with 3D-BN showed a distinct increase of TC on through-plane orientation with increasing sizes of BN. To be more specific, the through-plane TC of A-3D-SBN/epoxy was 1.10 W/m·K under 25 °C, while that of the A-3D-LBN/epoxy was up to 1.47 W/m·K, which is mainly attributed to the different intensity of the phonon transport. BN platelets with a larger lateral size (LBN) in the composite were easier to connect to each other and formed more thermal transport paths, and they led to fewer interfaces, resulting in less phonon scattering and lower interface thermal resistance. Moreover, the LBN in the composite had better alignment along the through-plane direction, which enabled the ultra-high TC in the (002) crystal plane of BN to improve the thermal conductivity. As mentioned above, LBN had a higher ratio of lateral size and thickness, leading to a preferential alignment during self-assembly processing. Compared to the composites prepared by atmospheric infiltrating, the composites prepared by vacuum infiltrating showed regular similar changing, while the through-plane TC of V-3D-BN/epoxy composites increased in increments of the BN sizes. For instance, V-3D-LBN/epoxy exhibited the highest through-plane TC of 2.01 W/m·K, which was an increase of 38% over that of V-3D-SBN/epoxy. Moreover, V-3D-LBN/epoxy showed a significant enhancement of the through-plane TC compared to A-3D-LBN/epoxy. It was mainly ascribed to the undamaged 3D network skeletons and fewer voids. In order to verify the conclusion, the TGA curves of the BN/epoxy composite prepared by different methods were measured, and the results are shown in Figure 3b. It should be noted that the BN loads in the epoxy composites were slightly different. For example, the volume fractions of A-3D-LBN/epoxy and V-3D-LBN/epoxy were 18.3% and 15.4%, respectively. The volume fraction of V-3D-LBN/epoxy was slightly low, indicating it had fewer voids.

The 3D network with oriented and interconnected structure can form more thermal transport channels and increase phonon transport and reduce interface thermal resistance. Compared with V-3D-LBN/epoxy, A-3D-LBN/epoxy showed lower TC due to the existence of voids and bubbles, of which the void percentage was calculated from the TGA results in Figure 3b. In addition, the other important factor is the collapse of the partial 3D network structure during degassing in the LBN/epoxy composite. Figure 3c shows the dependence of the TC of specimens on temperature. The TC of epoxy and its composites showed a similar variation tendency, which slightly increased at first and subsequently decreased with increasing test temperature. The TC slightly decreased to the temperature above the glass transition temperature of the samples. At this temperature, the specimens were softer leading to a deterioration of the thermal transport networks, reducing phonon transport [5,13,42]. Actually, the dependence of the TC of 3D-BN/epoxy composites on temperature was less. In other words, the V-3D-LBN/epoxy composite still showed the highest TC over all range of temperature because the BN with a bigger lateral size would have less interface and a higher orientation.

To investigate the effect of sizes of BN on its orientation, the directions and angles of BN in the composite were demarcated to quantitatively analyze the orientation degree of BN. The 90° and 0° represented perpendicular and parallel to sample surface, respectively. Based on some previous references [43,44], the degree of BN orientation in the epoxy could be obtained via XRD due to the different structure and diffraction peaks of BN in various crystal plane directions. Specifically, the in-plane alignment of BN in the composites should show a stronger peak intensity of (002) crystal plane and a lower peak intensity of (100) crystal plane. In other words, the peak intensity ratio of the (100) plane and (002) plane, appearing at approximately 26.8° and 41.7°, can represent the oriented degree of BN platelets in the through-plane direction. In addition, the degree of orientation increased with the increment of the ratio of (100) plane and (002) plane. The XRD curves and peak intensity ratios of BN/epoxy composites are exhibited in Figure 3d. The peak intensity ratio of V-3D-LBN/epoxy was approximately 0.42, which was obviously higher in comparison with V-3D-SBN/epoxy (0.26), indicating that the orientation degree of BN in the vertical plane direction obviously increased. The XRD results further prove that BN with a larger size in the epoxy composites will achieve better alignment during filtrating self-assembly.

### 3.3. Dielectric and Thermal Mechanical Properties

**Figure 4:**
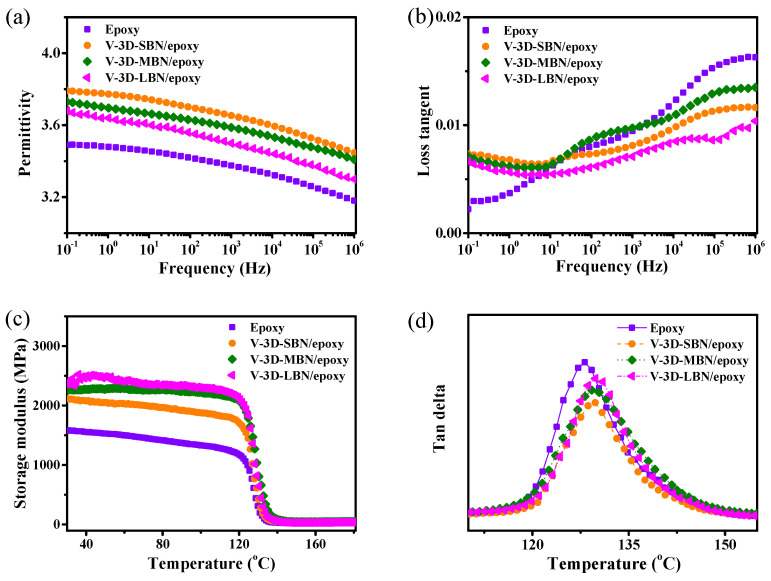
(**a**) Dielectric constant and (**b**) loss tangent of neat epoxy and the V-3D-BN/epoxy composites. DMA curves of specimens: (**c**) storage modulus and (**d**) tan delta.

Based on the importance of dielectric properties, glass transition temperature and mechanical characteristics for microelectronic packaging, these properties were investigated and evaluated for their practical applications. Figure 4a exhibits the frequency dependence of permittivity of epoxy and its composites. The permittivity of the epoxy composites slightly decreased as the frequency rose at room temperature due to the mismatch of turning-direction polarization and frequency change. The dielectric constant of BN/epoxy composites was a little higher than that of pure epoxy, although the intrinsic dielectric constant of BN was lower. It can be attributed to the interface’s polarization between submicron BN platelets (in thickness) and the epoxy molecular, increasing the dielectric constant of composites. Moreover, the dielectric response at a high frequency could be owed to the oriented polarization of the polymer molecular [45]. V-3D-SBN/epoxy showed slightly lower permittivity compared to V-3D-LBN/epoxy due to the greater thickness of LBN, leading to a more relaxed interface and an easier turning-direction polarization of the polymer molecular. Moreover, the V-3D-SBN/epoxy composite had more voids, of which the permittivity of the air was extremely low, resulting in a lower permittivity. The frequency dependence of dielectric loss of V-3D-SBN/epoxy, V-3D-MBN/epoxy and V-3D-LBN/epoxy composites is shown in Figure 4b. The dielectric loss of epoxy and its composites tended to increase gradually with increasing frequency. Compared to epoxy, the dielectric loss of composites below 10 Hz was higher due to the interface loss between BN and the epoxy matrix. But their loss lines were still quite low. For instance, the loss tangent of V-3D-LBN/epoxy was only 0.006 at a power frequency of 50 Hz.

Figure 4c,d shows the dynamic mechanical properties of epoxy and its composites at different temperatures. Epoxy composites have a much higher storage modulus compared to pure epoxy in Figure 4c, among which V-3D-LBN/epoxy showed the highest storage modulus due to the fewest voids and undamaged 3D networks in the composite. Moreover, the ladder-shaped decreases of the storage modulus were observed from 120 °C to140 °C due to the transition from a glassy state to a rubber state of the epoxy matrix. The loss factor (tan delta) of the samples is shown in Figure 4d, and the peaks of the loss factor can denote the glass transition temperature (Tg). It can be seen that the epoxy composites showed higher Tg due to the good thermal stability of BN and the constraining mobility of the epoxy molecular chains. Similar research results can be observed in [46,47,48]. For example, Uthaman et al. found a significant increase in the Tg of the composite after adding carbon fiber to the epoxy resin. Idrisi et al. found that the Tg of epoxy resin could be improved by combining alkali-free glass fiber with epoxy resin.

## 4. Conclusions

In this work, we presented a simple and eco-friendly strategy via filtrating self-assembly and vacuum infiltration to obtain the 3D-BN network skeleton with alignment in the through-plane direction, and we investigated the effect of BN sizes on through-plane TC in the epoxy matrix. Specifically, the through-plane TC values of A-3D-SBN/epoxy, A-3D-MBN/epoxy and A-3D-LBN/epoxy were 1.10, 1.24 and 1.47 W/m·K, respectively. BN with a bigger lateral size in the epoxy matrix was easier to connect to each other, leading to less phonon scatter. More importantly, a technique of epoxy pouring in a vacuum condition was developed to make bulk BN be fully filled the with epoxy solution. The V-3D-LBN/epoxy composite of 15.4 vol.% BN had a through-plane TC of 2.01 W/m·K, comparatively low permittivity of 3.7 and a loss tangent of 0.006 at 50 Hz, which is suitable for the application of microelectronic devices. This work shows that BN with larger transverse dimensions is more likely to improve thermal conductivity, so the transverse dimensions of two-dimensional materials should be emphasized and pursued in subsequent studies, rather than focusing only on obtaining thinner two-dimensional materials. It is significantly important for practical application in the next generation of microelectronic devices to study the effect of size of two-dimensional materials on thermal conductivity because it is not just related to performance, but also related to the cost of materials.

## Data Availability

Data is contained within the article.

## Supplementary Materials

The following supporting information can be downloaded at: https://www.mdpi.com/article/10.3390/nano12030446/s1, Figure S1: SEM images of h-BN powder with marked diameter (a) SBN, (c) MBN, (e) LBN. Figure S2: SEM images of h-BN powder with marked thickness (a) SBN, (c) MBN, (e) LBN; Thickness distribution of h-BN powder calculated by Guass equation (b) SBN, (d) MBN, (f) LBN.
