# Supplementary material for: The Investigation of the Effect of Filler Sizes in 3D-BN Skeletons on Thermal Conductivity of Epoxy-Based Composites
Source: Nanomaterials (Basel). 2022 Jan 28;12(3):446. doi: 10.3390/nano12030446 (PMC8839376; doi:10.3390/nano12030446)
Supplement: Supplementary file 1 [file nanomaterials-12-00446-s001.zip › nanomaterials-1536569-supplementary.pdf]

Supplementary Materials

The investigation of the effect of filler sizes in 3D-BN skeletons on thermal conductivity of epoxy-based composites

Zhengdong Wang [1,2,*], Tong Zhang [1], Jinkai Wang [1,2], Ganqiu Yang [1], Mengli Li [1] and Guanglei Wu [3,*]

[1] School of Mechanical and Electrical Engineering, Xi'an University of Architecture and Technology, Xi'an, 710055, China; zt@xauat.edu.cn (T.Z.); jkwang@xauat.edu.cn (J.W.); yangganqiu@xauat.edu.cn (G.Y.); limengli@xauat.edu.cn (M.L.)

[2] Shaanxi Key Laboratory of Nano Materials and Technology, Xi'an University of Architecture and Technology, Xi'an, 710055, China

[3] Institute of Materials for Energy and Environment, State Key Laboratory of Bio-fibers and Eco-textiles, College of Materials Science and Engineering, Qingdao University, Qingdao 266071, P. R. China;

* Correspondence: Correspondence: wangzhengdong@xauat.edu.cn (Z.W.); wuguanglei@qdu.edu.cn (G.W.)

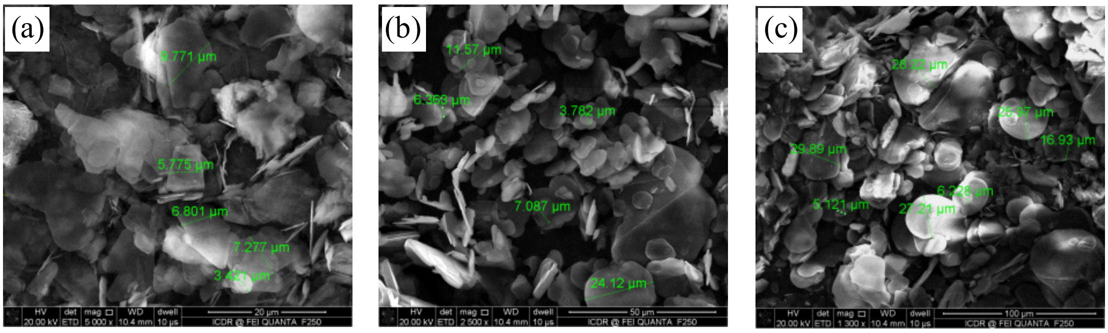

Figure S1. SEM images of h-BN powder with marked diameter (**a**) SBN, (**c**) MBN, (**e**) LBN.

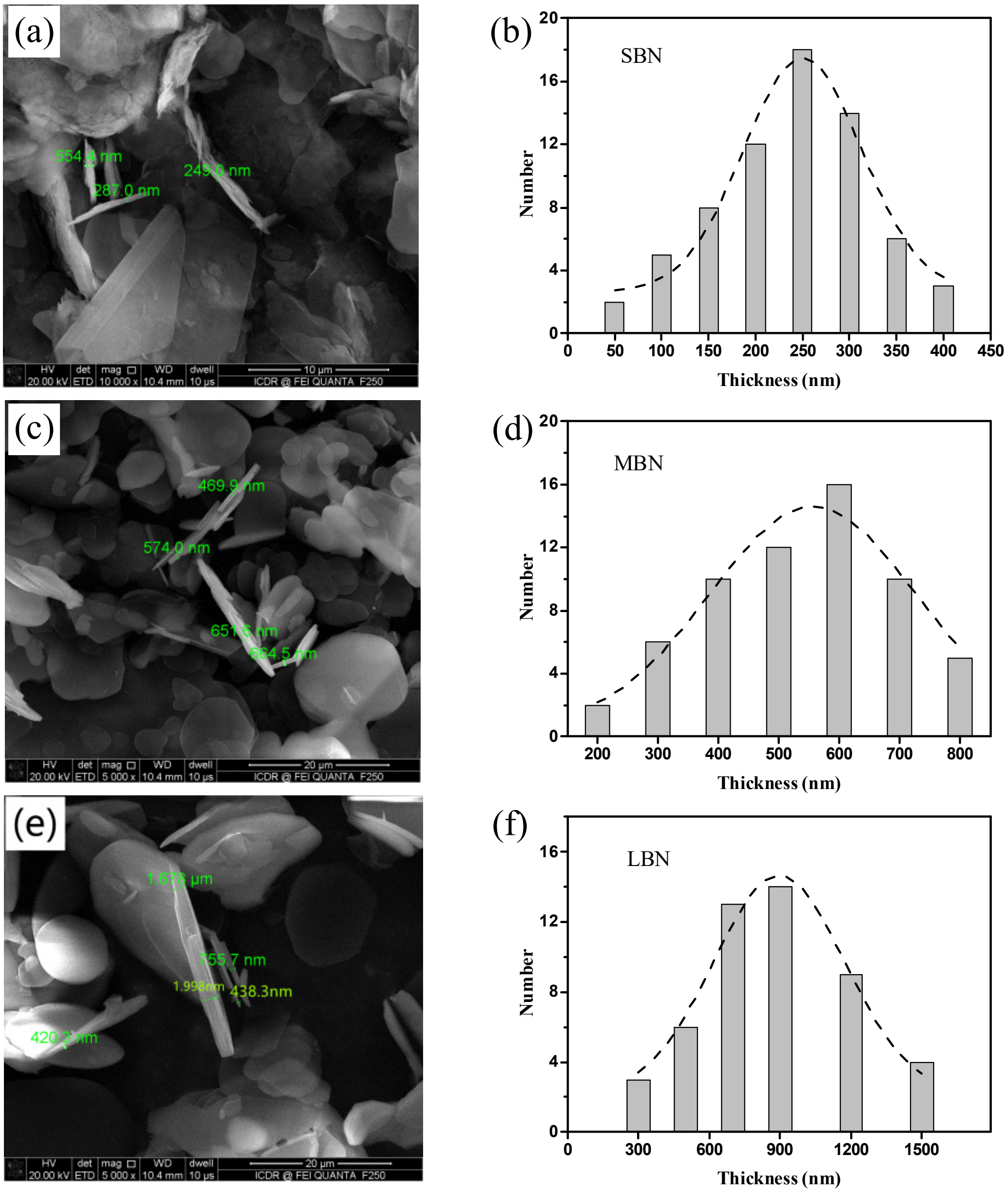

Figure S2. SEM images of h-BN powder with marked thickness (**a**) SBN, (**c**) MBN, (**e**) LBN; Thickness distribution ofh-BN powder calculated by Guass equation (**b**) SBN, (**d**) MBN, (**f**) LBN.
